# Testing the sensitivity and specificity of the fluorescence microscope (Cyscope^®^) for malaria diagnosis

**DOI:** 10.1186/1475-2875-9-88

**Published:** 2010-03-31

**Authors:** Saad El-Din H Hassan, Somia I Okoued, Mahmoud A Mudathir, Elfatih M Malik

**Affiliations:** 1 Emergency and Humanitarian Action Directorate, Federal Ministry of Health, Khartoum, Sudan; 2 Communicable Disease Control Directorate, State Ministry of Health, Kassala, Sudan; 3 National Malaria Control Programme, Federal Ministry of Health, Khartoum, Sudan; 4 Communicable Disease Control Directorate, Federal Ministry of Health, Khartoum, Sudan

## Abstract

**Background:**

Early diagnosis and treatment of malaria are necessary components in the control of malaria. The gold standard light microscopy technique has high sensitivity, but is a relatively time-consuming procedure especially during epidemics and in areas of high endemicity. This study attempted to test the sensitivity and specificity of a new diagnostic tool - the Cyscope^® ^fluorescence microscope, which is based on the use of Plasmodium nucleic acid-specific fluorescent dyes to facilitate detection of the parasites even in low parasitaemia conditions due to the contrast with the background.

**Methods:**

In this study, 293 febrile patients above the age of 18 years attending the malaria treatment centre in Sinnar State (Sudan) were interviewed using a structured questionnaire. Finger-prick blood samples were also collected from the participants to be tested for malaria using the hospital's microscope, the reference laboratory microscope, as well as the Cyscope^® ^microscope. The results of the investigations were then used to calculate the sensitivity, specificity, and positive and negative predictive values of the Cyscope^® ^microscope in reference to gold standard light microscopy.

**Results:**

The sensitivity was found to be 98.2% (95% CI: 90.6%-100%); specificity = 98.3% (95% CI: 95.7% - 99.5%); positive predictive value = 93.3% (95% CI: 83.8% - 98.2%); and negative predictive value = 99.6% (95% CI: 97.6% - 100%).

**Conclusions:**

In conclusion, the Cyscope^® ^microscope was found to be sensitive, specific and provide rapid, reliable results in a matter of less than 10 minutes. The Cyscope^® ^microscope should be considered as a viable, cheaper and time-saving option for malaria diagnosis, especially in areas where *Plasmodium falciparum *is the predominant parasite.

## Background

Malaria is a leading cause of morbidity and mortality in Africa, killing a child every 30 seconds [1]. One of the major contributing factors to malaria mortality is delayed or inaccurate diagnosis [2]. That is why one of the main strategic directions of the Roll Back Malaria strategic plan for Sudan, early diagnosis and treatment of malaria, is a necessary component in the control of malaria [3]. Early diagnosis has become even more important after the emergence of drug resistance [4].

In many countries, due to poverty or lack of training and facilities, malaria is still being diagnosed clinically, an unreliable method leading to over-diagnosis and wasting precious time for patients and staff [5]. The gold standard in malaria diagnosis is still light microscopy [6]. This technique has high sensitivity but is a relatively time-consuming procedure especially during epidemics and in areas of high endemicity. It requires trained experienced personnel and careful preparation and application of reagents to ensure quality results [7,8].

Many diagnostic procedures have been developed which aim at reducing the time, preparation, and training needed to diagnose malaria. The use of Plasmodium nucleic acid-specific fluorescent dyes was found to facilitate detection of the parasites even in low parasitaemia conditions due to the contrast with the background [9]. One recent addition is the advent of the Cyscope^® ^microscope produced by the German company Partec. This is a mobile, battery-operated microscope. The slides of the microscope come ready with malaria parasite DNA specific staining reagents in dried form. All that is needed is the addition of a drop of blood and viewing the slide under the microscope, saving time and preparation. An optional add-on enables viewing the slides on a computer to facilitate the diagnostic procedure and storage and retrieval of results [10].

Given the fact that the test is relatively cheap, this technique offers the possibility of a useful test especially for malaria endemic regions. This study was conducted out to investigate the sensitivity and specificity of the Cyscope^® ^microscope in reference to the gold standard of light microscopy.

## Methods

### Study area/setting

Sinnar state lies 270 km south-east Khartoum, the capital. With an average annual rainfall of 700-1000 mm, the prevalence of malaria in Sinnar state is 57/1,000 population. The malaria control programme in Sinnar State Ministry of Health is one of the best state programmes building on the history of excellence as the state during eradication era was selected for piloting eradication in Sudan. Moreover, Sinnar state hosted since early 1960s, the malaria training centre with good reputation. The centre nowadays acts as a centre for training malaria microscopists all over Sudan and also used by the national malaria control programme as a venue for short courses in epidemiology, vector control and planning for malaria.

### Study design

This is a cross-sectional facility-based analytical study

### Study subjects

All patients with fever, over 18 years of age reporting to the malaria centre (Sinnar Hospital) were included in the study with exclusion of anyone who is receiving or has received malaria treatment within 14 days prior to the study and severely sick patients.

### Sample size

For the calculation of the sample size, we used the following equation [11]:

Where:

N = sample size, the suspected sensitivity (p) is 0.90 and the minimal acceptable sensitivity (p_0_) is 0.75.

This translates into N = 42. Among the patients in the study who are all febrile, the prevalence of malaria is expected to be not less than 20%. Therefore, to adjust the sample size for this factor, the calculated sample size (42) was multiplied by 5 to get a sample size of 210. The sample size adjustment for specificity yielded a much smaller sample (approx 52). The sample size used in the study was 293.

### Sampling technique

All patients attending the site were included until the sample size was reached.

### Data collection

A structured data collection form was used to record the results of the light and fluorescence microscope. After verbal informed consent was obtained, the data collection form was filled out through direct interview with the patients. The form focused on basic personal data and was used later to record the results of light and fluorescence microscopy. After the form is filled out, two finger-prick blood samples were taken; one for light microscopy, and the other for Cyscope^® ^microscopy.

For the light microscopy, a light microscope was used to diagnose malaria using thick and thin film, by a trained person blinded to the results of the Cyscope^® ^microscope. Thin and thick films were stained using 10% Giemsa stain. Hundreds of fields were examined at 10 × 100 magnification under oil immersion for the presence of trophozoite and gametocyte stages, and for identification of parasite species. The density was estimated by counting the number of asexual parasites per 200 white blood cells and then multiplied by 40 to get the parasite number per 8,000 white blood cells.

A Cyscope^® ^malaria microscope was used to diagnose malaria by another trained person, blinded to the results of light microscopy. According to Partec [10], a finger-prick blood sample was collected from each case, after informed consent was obtained. This sample was applied to the pre-prepared slides of the fluorescence microscope to be viewed with the microscope. Malaria was confirmed by viewing the fluorescent DNA of plasmodia under the microscope (objective × 40). The parasites were viewed on a computer which was connected to the microscope digital camera.

### Data management and analysis plan

Epi Info (Version 3.5.1 - 13 August 2008) was used for data entry, data cleaning, and analysis. Quantitative data was summarized using proportions and means. With the results from the light microscope as the standard, sensitivity of the Cyscope^® ^was calculated as true positives/(true positive + false negatives), specificity as true negatives/(true negatives + false positives), positive predictive value (PPV) as true positives/(true positives + false positives), negative predictive value (NPV) as true negatives/(true negatives + false negatives).

### Verbal consent

Verbal consent was taken from each participant in the study.

## Results

The number of patients included in the study was 293. The mean age was 32.5 years (min: 18; max: 90; STD: 13.43). Females constituted 60.7% (n = 176) of the population studied. Of these, 19.8% (n = 33) were pregnant) (see Table 1). The reference lab results showed that 19.5% (n = 57) of those tested were positive for malaria (all *P. falciparum*) (see Table 2). The parasite count in all positive slides was above 100/μl (mean: 7,849 parasites/μl). The Cyscope^® ^fluorescence microscope results showed that 20.5% (n = 60) of those tested were positive for malaria.

**Table 1:** Summary of characteristics of study population and results, Sinnar State, Sudan, 2009

Characteristic	Result
**Age**	

Mean Age	32.5 years (SD: 13.4)

Median Age	30 years

**Gender**	

Female:Male ratio	1.54:1

Percentage of Females Pregnant	19.8%

**Education**	

Basic/undergraduate	117 (40.9%)

University/post-graduate	89 (31.1%)

No formal education	80 (28%)

**Occupation**	

House wife	135 (47.4%)

"Free" trade	50 (17.5%)

Employee (Public Sector/Private Sector/Organization)	25 (8.8%)

Unemployed	75 (26.3%)

**Table 2:** Cyscope^® ^fluourescence microscope results compared to Reference Laboratory Light Microscopy Results, Sinnar State, Sudan, 2009

		Cyscope^® ^Result		
		
		Positive	Negative	Total
**Reference Lab Result**	**Positive**	56	1	**57**
	
	**Negative**	4	232	**236**

**Total**		**60**	**233**	**293**

Sensitivity: 98.2% (95% CI: 90.6%-100%)

Specificity: 98.3% (95% CI: 95.7% - 99.5%)

Positive Predictive Value: 93.3% (95% CI: 83.8% - 98.2%)

Negative Predictive Value: 99.6% (95% CI: 97.6% - 100%).

Parasite count: Mean (to the nearest whole number): 7849 parasites/μl (SD: 10897); Median: 1960 parasites/μl; Mode: 160 parasites/μl

Of those tested positive by the reference laboratory, 56 have been found to be positive by the Cyscope^® ^fluorescence microscope while one was negative (see Table 2). This translates into a sensitivity of 98.2% (95% CI: 90.6%-100%). Of those samples, which tested negative by the reference laboratory, 232 were negative, while four were positive by the Cyscope^® ^fluorescence microscope. This translates into a specificity of 98.3% (95% CI: 95.7% - 99.5%).

Of those tested positive by the Cyscope^® ^fluorescence microscope, 56 were positive by the reference laboratory, while four were negative. This translates into a positive predictive value of 93.3% (95% CI: 83.8% - 98.2%). Of those tested negative by the Cyscope^® ^fluorescence microscope, 232 were negative according to the reference laboratory while one was positive. This translates into a negative predictive value of 99.6% (95% ci: 97.6% - 100%).

## Discussion

The females in the sample were slightly more than the males. This might have accounted for having 33 pregnant women in the sample, two of whom were positive for malaria. As expected, the prevalence of malaria within the sample was around 20%. All positive samples were *P. falciparum*. This was also expected since *P. falciparum *is by far the most dominant species in Sudan [3].

The accepted level of sensitivity for a rapid diagnostic test in diagnosing malaria is a sensitivity of 95% at a parasite density of 100 parasites/μl [12]. In this study, the results of the reference laboratory were very similar to those of the Cyscope^® ^fluorescence microscope. The calculated sensitivity of the Cyscope^® ^fluorescence microscope in diagnosing malaria was above the 95% threshold while the specificity was above the 90% threshold [7]. The positive predictive value and the negative predictive value of the Cyscope^® ^fluorescence microscope were also high (above 90%) in comparison to the gold standard of light microscopy in the reference laboratory.

## Conclusions

In conclusion, this study has found the Cyscope^® ^fluorescence microscope to be a reliable diagnostic tool that is very sensitive and specific in diagnosing falciparum malaria. Since this is the predominant species in Sudan, and is the species causing most mortality and complications, this is very relevant and very useful. It is expected that the microscope will show similar results for other malaria species, but this could not be ascertained by this study. Further studies are needed to determine effectiveness in diagnosing other Plasmodium species. Further studies are also needed to determine the effectiveness of the Cyscope^® ^fluorescence microscope in diagnosing malaria in cases of low parasite densities.

## Competing interests

The authors declare that they have no competing interests.

## Authors' contributions

HSH participated in the design and coordination of the study and performed the statistical analysis. OSI participated in coordinating the study. MMA participated in coordinating the study in Sinnar State. MEM conceived of the study, and participated in its design and coordination and helped to draft the manuscript. All authors read and approved the final manuscript.
